# Magnetically-guided, stimuli-responsive microdevices for endovascular therapy

**DOI:** 10.1007/s10544-026-00791-4

**Published:** 2026-01-20

**Authors:** Sanjay Manoharan, Vivek Subramanian

**Affiliations:** Laboratory for Advanced Fabrication Technologies, Institute of Electrical and Micro Engineering, École Polytechnique Fédérale de Lausanne (EPFL), Neuchâtel, 2000 Switzerland

**Keywords:** Microrobots, Flow disruption, Aneurysm, Endovascular devices, Magnetic actuation, Stimuli responsive materials

## Abstract

Current endovascular aneurysm treatments rely on catheter-based delivery systems, which inherently restrict access to tortuous anatomies and small-caliber vessels. To address this limitation, we introduce a tetherless microdevice platform that combines magnetic guidance with near-infrared (NIR) triggered shape-memory polymer (SMP) deployment for wireless aneurysm therapy. In this system, an external actuator magnet steers a microdevice-integrated effector magnet through anatomically realistic silicone vascular phantoms, while melanin-doped PLA structures enable precise NIR-induced shape recovery. Because NIR light penetrates biological tissue, deployment can be activated non-invasively from outside the body. The platform supports two device architectures tailored to different clinical needs: a spiral flow disruptor with a retrievable magnet for partial inflow modulation and a petalloid occluder designed for permanent sealing of narrow-neck aneurysms. Their navigation behavior was modelled using a flow-responsive, magnetically modulated stick–slip (F-MMSS) framework that captures the influence of pulsatile flow and wall interactions. Experimentally, magnetic steering was demonstrated under physiologically relevant flow rates and NIR activation achieved reliable deployment through ex vivo tissue. Particle image velocimetry and computational fluid dynamics confirmed substantial reductions in intra-aneurysmal velocity across multiple geometries. Material characterization further verified that PLA–melanin composites exhibit suitable bio and hemocompatibility for preliminary use. Together, these results establish a proof-of-concept platform for wireless navigation and remote deployment of endovascular microdevices, motivating future in vivo evaluation.

**Graphical abstract:**

Schematic illustration of a magnetically guided, NIR-triggered microdevice enabling targeted deployment of shape-memory occluders for aneurysm and tumor vessel therapy.

**Figure:**
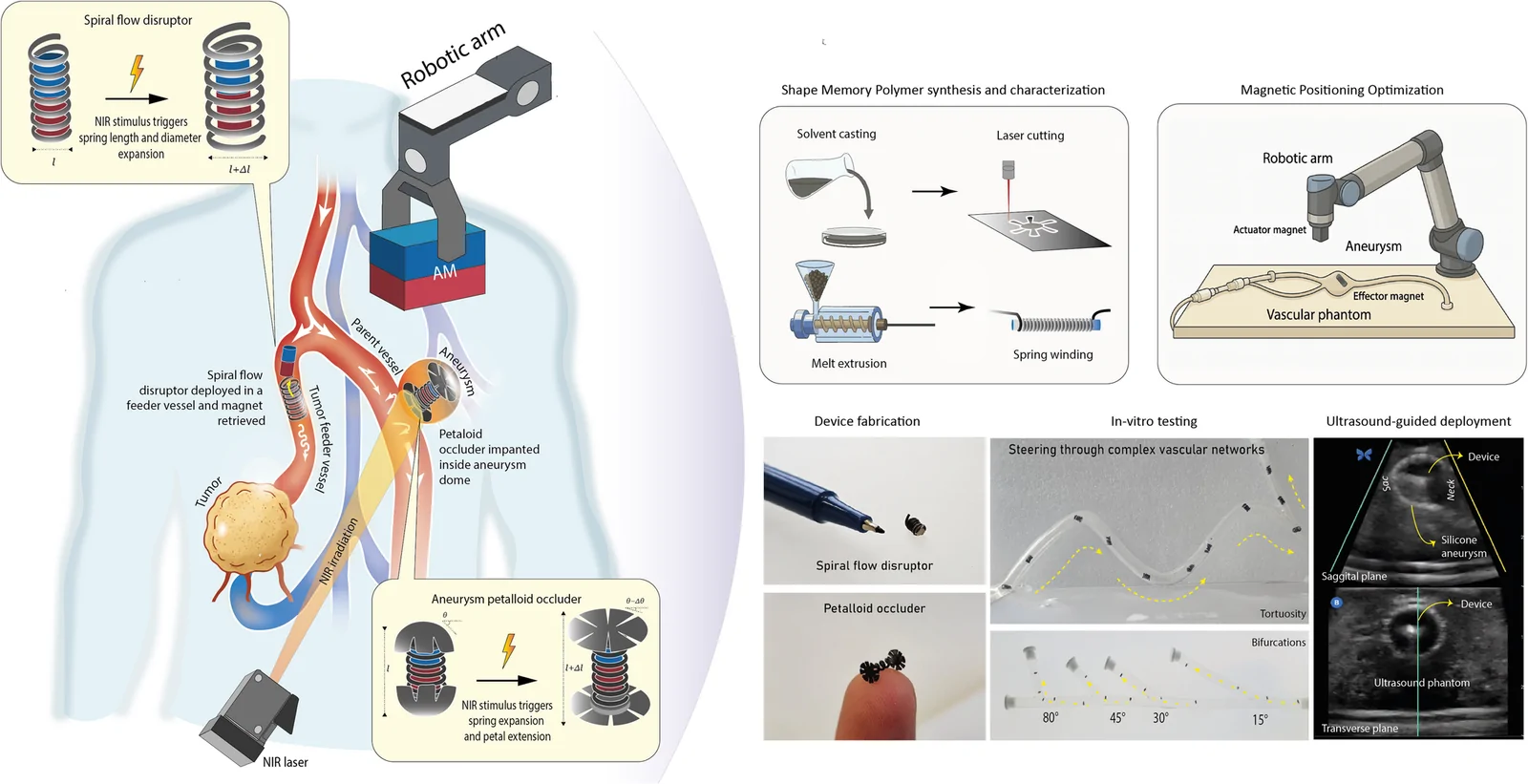

**Supplementary Information:**

The online version contains supplementary material available at https://doi.org/10.1007/s10544-026-00791-4.

## Introduction

Endovascular diseases such as aneurysms, arteriovenous malformations and vascular tumors often require targeted interventions via blood vessels. Traditional treatments typically involve catheter-based delivery of coils [1], stents [2], or embolic agents [3] which are effective but limited by the workspace constraints of tethered access [4]. These procedures rely on tethers such as catheters or guidewires to provide control and imaging power delivery. These tethers inherently limit degrees of freedom, reduce maneuverability and elevate the risk of tissue injury or infection [5–7] Beyond such catheter-deployed coils and flow diverters, other active endovascular technologies also highlight existing limitations. Shape-memory-alloy (SMA) stents provide reliable thermomechanical expansion but remain fully catheter-dependent and cannot be reconfigured once deployed [8, 9]. Magnetically steerable guidewires and catheters improve access in tortuous anatomy yet still rely on a physical tether and therefore inherit the workspace and friction constraints of conventional endovascular tools [10, 11]. Magnetic microrobots, meanwhile, demonstrate true untethered navigation, but are primarily locomotion-only systems lacking deployable or shape-changing structures and often require large electromagnetic setups [12–14]. As a result, the development of tetherless therapeutic devices represents a natural evolution in endovascular intervention, enabling navigation and treatment in hard-to-reach sites with tortuous anatomy. Here, we propose such an approach using a combination of wireless magnetic control and stimuli-responsive deployment using Near-Infrared (NIR) light.

NIR-triggered shape memory polymers have garnered significant interest for non-invasive, on-dem and therapeutic and diagnostic deployment within physiological environments. For instance, NIR-responsive SMP stents and contraceptives were implanted with excellent biocompatibility in rodent models [15–18]. Crucially, this exploited deep tissue penetration (700–1000 nm) of NIR light to trigger photothermal transition from temporary to permanent shape without invasive heating. Despite such promise, existing NIR-triggered devices rely on tethered or surgically implanted platforms, which undermine the inherent advantage of wireless triggering enabled by remote NIR stimulation and limit their clinical translation. To fully realize the benefits of such stimuli-responsive systems, there is a clear need to combine them with tetherless positioning capabilities.

Various wireless positioning strategies, such as acoustic, optic and magnetic manipulation, have been explored to achieve tetherless position [19, 20]. Among these, magnetic control, particularly with permanent magnets, offers strong and adjustable field strengths with no need to supply external power and minimal biological attenuation, ideal for intravascular use [12]. Magnetically-guided microbots in prior implementations have usually combined magnetic actuation with other non-magnetic forces or using complex multi-axis magnetic fields [21, 22]. In contrast, recent studies have demonstrated wireless navigation of untethered magnetic robots via rotating magnetic fields in an exvivo vasculature model [23]. However, focus has remained solely on locomotion, without analyzing the details of interactions with biological fluid flow. On the other h and, current endovascular microrobots have demonstrated therapeutic capabilities such as drug delivery based on passive elution [12, 24] or mechanical breakdown of clots via magnetic fields [25, 26], but lack morphological transformation capabilities. In this work, we demonstrate and optimize a simplified Actuation Magnet–Effector Magnet (AM–EM) setup that operates with only two degrees of freedom yet enables a surface-mediated, ceiling-constrained positioning using a step-slide gait. We use this robot in conjunction with NIR-triggered shape memory polymer to demonstrate triggered morphological transformation.

**Fig. 1:**
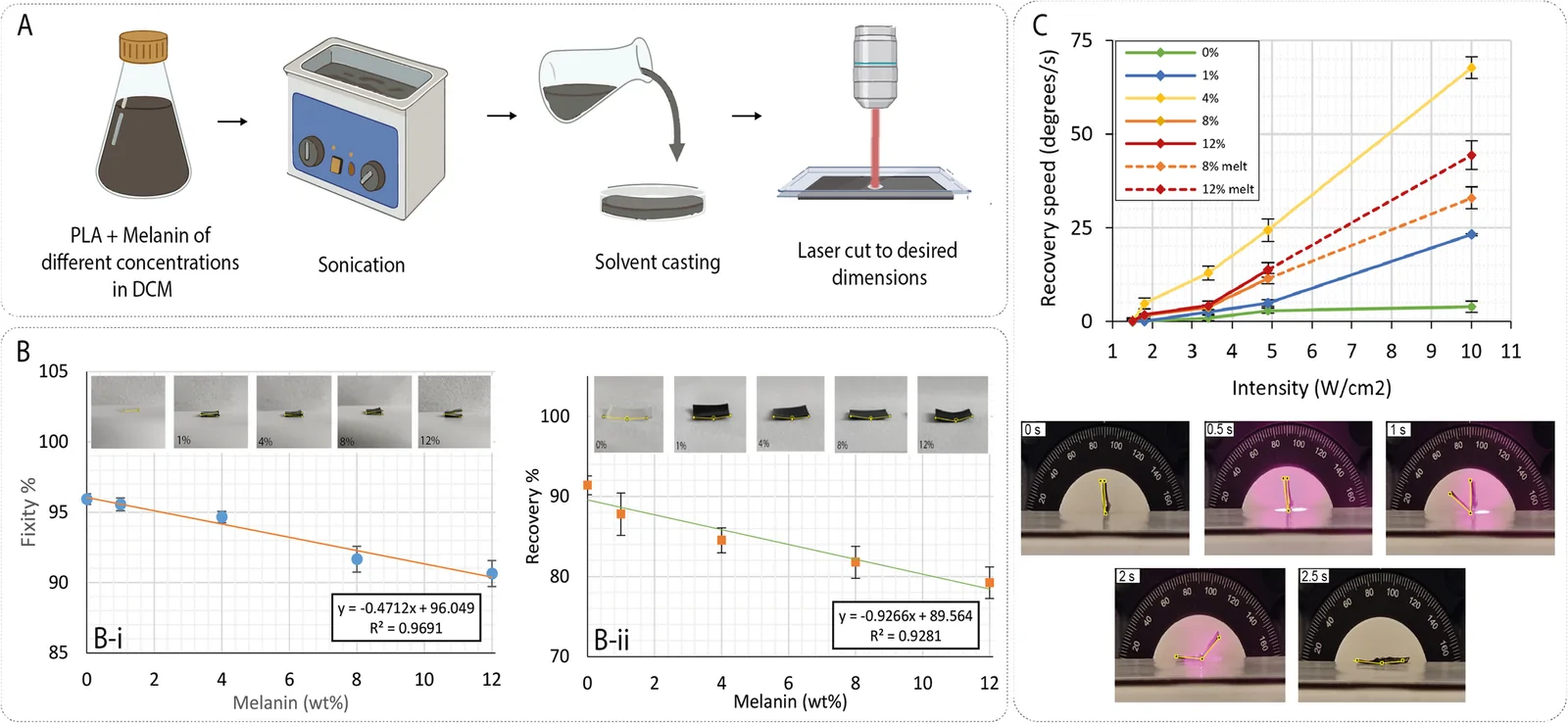
NIR-Responsive Shape Memory Polymer (SMP) beam characterization.** (A)** Workflow for PLA–melanin film preparation via solvent casting at varying melanin concentrations. **(B)** Beam recovery: (i) Fixity decreases with increasing melanin loading; (ii) recovery percentage also declines with melanin content; (iii) Photothermal conversion efficiency of PLA-melanin as a function of melanin concentration **C)** Angular recovery speed increases with NIR intensity, particularly at low-to-moderate melanin levels; and its corresponding images. Statistical analysis was based on triplicate measurements (*n* = 3) for all experiments except where explicitly stated. Data points show mean ± standard deviation

This study thus addresses key gaps in wireless endovascular devices - most magnetic microrobots lack morphological transformation capabilities, NIR-triggered shape memory systems lack wireless positioning and no platform has integrated both to enable morphological transformation along with magnetic navigation. Here, we present a novel approach combining magnetically guided microdevice for endovascular flow disruption and occlusion, combining magnetic navigation with NIR-triggered deployment. A two-magnet configuration - an external Actuation Magnet (AM) and an onboard Effector Magnet (EM), enables well-controlled ceiling-constrained vessel wall navigation. The deployable structure, made from a biocompatible PLA-melanin composite, undergoes photothermal shape recovery upon NIR exposure which penetrates the tissue (700–900 nm) [27, 28], allowing non-invasive activation without onboard electronics even in anatomically challenging vascular sites. This work thus represents the first proof-of-concept demonstration of NIR-responsive shape memory deployment guided by magnetic actuation for wireless endovascular therapy (Fig. 1). Unlike coiling, flow diverters, intrasaccular devices, or surgical clipping, this platform targets a distinct niche: distal vessels beyond catheter reach. Established modalities offer strong clinical validation but remain constrained by tethered delivery. Our tetherless system instead provides navigation freedom in small-calibre, angulated anatomies where catheter-based methods lose effectiveness (Supp Table. S3).

Two device architectures were explored to achieve distinct therapeutic goals. The first, a petaloid occluder, is designed for complete aneurysm neck sealing. It consists of a central magnet enclosed by a spring with petal-like arms at both ends. Upon NIR activation, the spring elongates and the petals unfold to conform tightly around the aneurysm neck and the top of the dome, blocking inflow and promoting thrombosis and complete sealing. The second, a spiral disruptor, is intended for flow modulation and features a magnet encased in a spring that expands in both diameter and length upon triggering. This allows it to coil along the vessel or aneurysm wall, inducing turbulence and partial inflow reduction. Even partial inflow reduction can extend residence time and promote thrombosis, especially in high aspect ratio aneurysms [29–32]. It is also suited for targeting tumor-feeding vessels, where flow reduction rather than full occlusion is desired. After deployment, the spiral polymer scaffold remains anchored while the embedded magnet can be retrieved, unlike for the occluder, in which the magnet remains implanted. These architectures thus address complementary clinical scenarios: occluders are designed for narrow-neck aneurysms (≤ 5 mm) where compact pre-deployment dimensions enable navigation into the constrained physical environment of the aneurysm and spiral disruptors target wide-neck geometries where complete occlusion is challenging and magnet retrieval in confined spaces become impractical.

Both devices are magnetically navigated in compact form and triggered via externally applied NIR light to recover their functional shapes via triggered morphological transformation. Flow disruption was validated using Particle Image Velocimetry (PIV) and Computational Fluid Dynamics (CFD), with the occluder achieving > 75% intra-aneurysmal velocity reduction and the disruptor showing successful partial suppression with redirected flow. Migration force testing confirmed > 3× higher retention for the occluder. Hemocompatibility and degradation assays confirmed minimal hemolysis and fouling, suggesting future clinical translation for tetherless endovascular therapy.

## Materials and methods

### Materials and device fabrication

Poly (lactic acid) (PLA, MW ~ 150,000) was blended with synthetic melanin (0–12 wt%). Films were prepared by solvent casting from dichloromethane (DCM) and dried under ambient and vacuum conditions. Extruded 1.75 mm PLA–melanin filaments were produced at 180–200 °C for use in spring and disruptor fabrication [33]. NdFeB permanent magnets were used as effector magnets (EM; cylindrical, 2 mm × 5 mm) and actuator magnets (AM; cube, 38–80 mm edge length). Both axial and diametric magnetizations were evaluated depending on navigation requirements. Petaloid occluders were fabricated by laser-cutting PLA–melanin films into petals, assembling them around a central NdFeB magnet and shape-programming the spring. Spiral disruptors were produced by winding PLA–melanin filaments around cylindrical magnets and programming them into compacted forms. Both constructs were designed to exp and to their functional shapes upon NIR triggering.

### Shape memory characterization

Differential scanning calorimetry (DSC) and thermogravimetric analysis (TGA) were used to determine glass transition and thermal stability from 25 to 600 °C at 10 °C/min under nitrogen. Tensile properties were measured on ASTM D638 Type IV specimens using a universal testing machine at 5 mm/min to calculate Young’s modulus. Shape fixity (Rf) and recovery (Rr) ratios were quantified using standard deformation–programming–recovery cycles. Recovery kinetics were assessed under 808 nm NIR irradiation (0.5–2 W/cm²) with thermal monitoring.

### Magnetic actuation system

A robotic arm positioned the AM over silicone vascular phantoms connected to a closed-loop pulsatile flow circuit (0–1000 mL/min; 60–150 bpm) to simulate physiological hemodynamics. Phantoms with varying geometries (straight, bifurcations, tortuosities) and aneurysm sac-to-neck ratios (10:10, 10:7.5, 10:5 mm) were used. Wall hardness ranged from Shore 30–60 A to mimic vessel compliance. Effector magnet motion was quantified by lateral offset (OF), step distance (SD) and positional error (PE) from tracked AM–EM trajectories. PE was computed using standard z-score comparison of mean positional distributions.

### Design of experiments (DOE)

A fractional factorial design screened 12 parameters, including AM size, EM weight, coupling distance, AM velocity, magnetization direction, pumping rate, viscosity, lumen hardness, friction coefficient and flow direction. Significant factors (identified via LogWorth scores) were validated using one-factor-at-a-time experiments (*n* = 3).

### Hemodynamic analysis

Velocity fields in aneurysm phantoms were obtained using fluorescent microspheres in a blood-analogue fluid. Images were recorded at 240 fps and processed using adaptive correlation algorithms in PIVLab [34]. CFD simulations were performed in COMSOL using laminar Navier–Stokes equations. Blood was modeled as a Newtonian fluid (ρ = 1060 kg/m³; µ = 3.5 mPa·s). Mesh refinement near walls used 0.1 mm elements. Mean intrasaccular velocity and vorticity were calculated from circular regions of interest encompassing the aneurysm sac and averaged over three cardiac cycles. Suppression was quantified as ((baseline – post-deployment)/baseline) × 100%.

### NIR triggering and deployment

Devices were activated using an 808 nm laser (0.5–10 W/cm²). Tissue penetration and deployment studies were performed through 1 cm of ex vivo tissue. Deployment was considered successful upon > 80% shape recovery within 60 s.

### Biocompatibility and safety assessment

Hemolysis assays followed ASTM F756-17. Samples were incubated with citrated whole blood and hemoglobin release was quantified at 540 nm relative to positive and negative controls. Cytotoxicity was assessed via MEM elution assays (ISO 10993-5) using NIH 3T3 fibroblasts exposed to material eluates for 48 h. Accelerated degradation was evaluated in 30% H₂O₂ at 37 °C. Samples were weighed periodically over 60 days and imaged for morphological changes. Deployed devices were subjected to increasing axial force until dislodgement using a custom tensile setup. Maximum displacement force was reported as migration resistance.

### Statistical analysis

All experiments were performed in triplicate (*n* = 3) unless otherwise stated. Data are reported as mean ± standard deviation. Extended experimental procedures, fabrication details and characterization protocols are provided in the Supplementary Materials.

## Results and discussion

This section presents the stepwise development and validation of a magnetically navigable, NIR-triggered microdevice for endovascular therapy. We begin with development and characterization of an NIR-triggered shape memory polymer, optimize magnetic guidance, combine both and fabricate deployable structures, in-vitro test their hemodynamic effects post-deployment and finally evaluate mechanical retention, biocompatibility and degradation.

### NIR-Responsive SMP performance

The first critical component of our integrated platform is the stimulus-responsive material that enables on-dem and shape transformation. To achieve reliable NIR-triggered deployment in vascular environments, we systematically optimized PLA-melanin composites for both photothermal efficiency and mechanical performance. Shape memory beams and springs were fabricated from Poly lactic acid (PLA)–melanin composites, with melanin selected as the photothermal additive since it facilitates light absorption in the near infrared, causing local heating and thus triggering of the PLA shape memory. Melanin concentrations ranging from 0 to 12 wt% were studied. Supplementary thermal and mechanical characterizations confirmed thermal stability up to ~ 230 °C across all formulations, with differential scanning calorimetry (DSC) and thermogravimetric analysis (TGA) showing no significant degradation. Tensile testing revealed that Young’s modulus increased markedly with melanin loading, indicating enhanced stiffness (Supp Fig. S1A, B, C). Photothermal conversion efficiency increased systematically with melanin loading, achieving temperature rises of 35–65 °C under NIR irradiation, with optimal heating balanced against mechanical processability at 6 wt% (Supp Fig. S2-A).

Shape recovery was evaluated by characterizing the same in pre-programmed laser-cut beams (Fig. 1A). Both fixity and recovery percentages decreased slightly with increasing melanin content (Fig. 1B-i, ii), indicating a trade-off between actuation efficiency and rigidity. However, all formulations-maintained fixity above 90% and recovery above 82%. Angular recovery speeds increased with laser intensity and remained robust up to 8 wt% melanin (Fig. 1C). Under a static load of ~ 500 mg, as a representative intraluminal mechanical challenge, chosen to approximate the forces an SMP beam might experience during deployment [35, 36], the bent beams recovered effectively upon NIR exposure, with output forces rising with melanin concentration (Supp. Fig.S2 -B, Supp.Vid. S1).

**Fig. 2:**
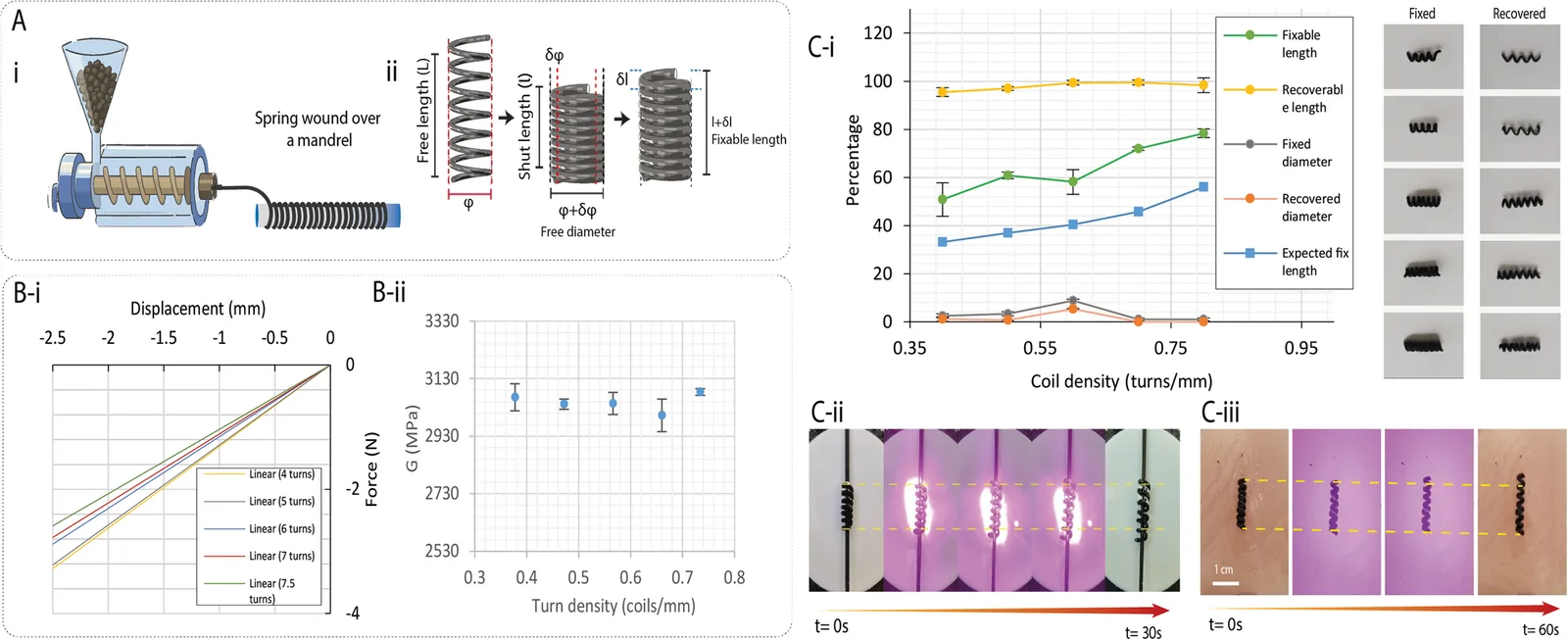
NIR-Responsive Shape Memory Polymer (SMP) spring characterization.** A)** Spiral spring fabrication: (i) PLA–melanin filament melt-extruded and wound on a mandrel; (ii) schematic of spring shape programming and NIR-triggered recovery, axial compression during programming induces slight radial expansion (ϕ + δϕ) and upon NIR exposure, axial recovery (l + δl) occurs with minimal diameter change, enabling geometric quantification; (iii) representative images of fixed vs. recovered spring geometry. **B)** Spring characterization: (i) Force–displacement curves for different turn counts; (ii) shear modulus *(G)* remains constant across densities using the spring constant relation; (iii) quantification of shape metrics vs. coil density; (iv-v) time-lapse images of NIR-induced spring recovery in a liquid medium and over 2.5 cm of tissue respectively. **C)** Spring characterization: **(i)** quantification of shape memory spring metrics vs. coil density; **(ii-iii)** time-lapse images of NIR-induced spring recovery in a liquid medium and over 1 cm of tissue respectively

Spiral springs were fabricated by winding extruded PLA–melanin filaments (Fig. 2A-i). Based on extrusion limits and photothermal-mechanical trade-off, 6 wt% melanin was selected for all spring-based experiments. Force–displacement tests revealed a near-linear elastic response with peak compressive forces of ~ 3.5 N (Fig. 2B-i). Spring constants yielded stable shear modulus values across varying coil densities (Fig. 2B-ii). During programming into a compacted configuration, modest radial expansion occurred (ϕ → ϕ + δϕ), which reversed upon recovery (Fig. 2A-ii). Fixable length (l) increased with decreasing spring thickness, while recoverable length (l + δl) remained consistent. These shape changes were quantitatively mapped (Fig. 2C-i). Upon NIR exposure, axial recovery was uniform across geometries, including successful activation through 1 cm of ex-vivo muscle at 10 W/cm² (350 J/cm² fluence), validating feasibility for trans-tissue deployment (Fig. 2C-ii, iii Supp.Vid. S1).

### Magnetic positioning and Flow-responsive, magnetically modulated Stick-Slip (F-MMSS)

#### The navigation challenge: controlling microrobots in pulsatile flow

With shape memory properties established, the next challenge was developing a reliable positioning system to deliver these devices to target sites within complex vascular anatomy. Intravascular navigation faces unique challenges: pulsatile blood flow creates time-varying forces that can cause device drifting or slipping, potentially leading to misplacement or downstream embolization. To address this, we developed a controlled locomotion strategy using magnetic forces imposed on an effector magnet (EM) embedded within the robot by an externally applied magnetic field. By exploiting the sliding behavior of the robot on vascular walls, we achieve precise positioning despite the limited degrees of freedom offered by a single external magnet.

#### Experimental platform and navigation metrics

**Fig. 3:**
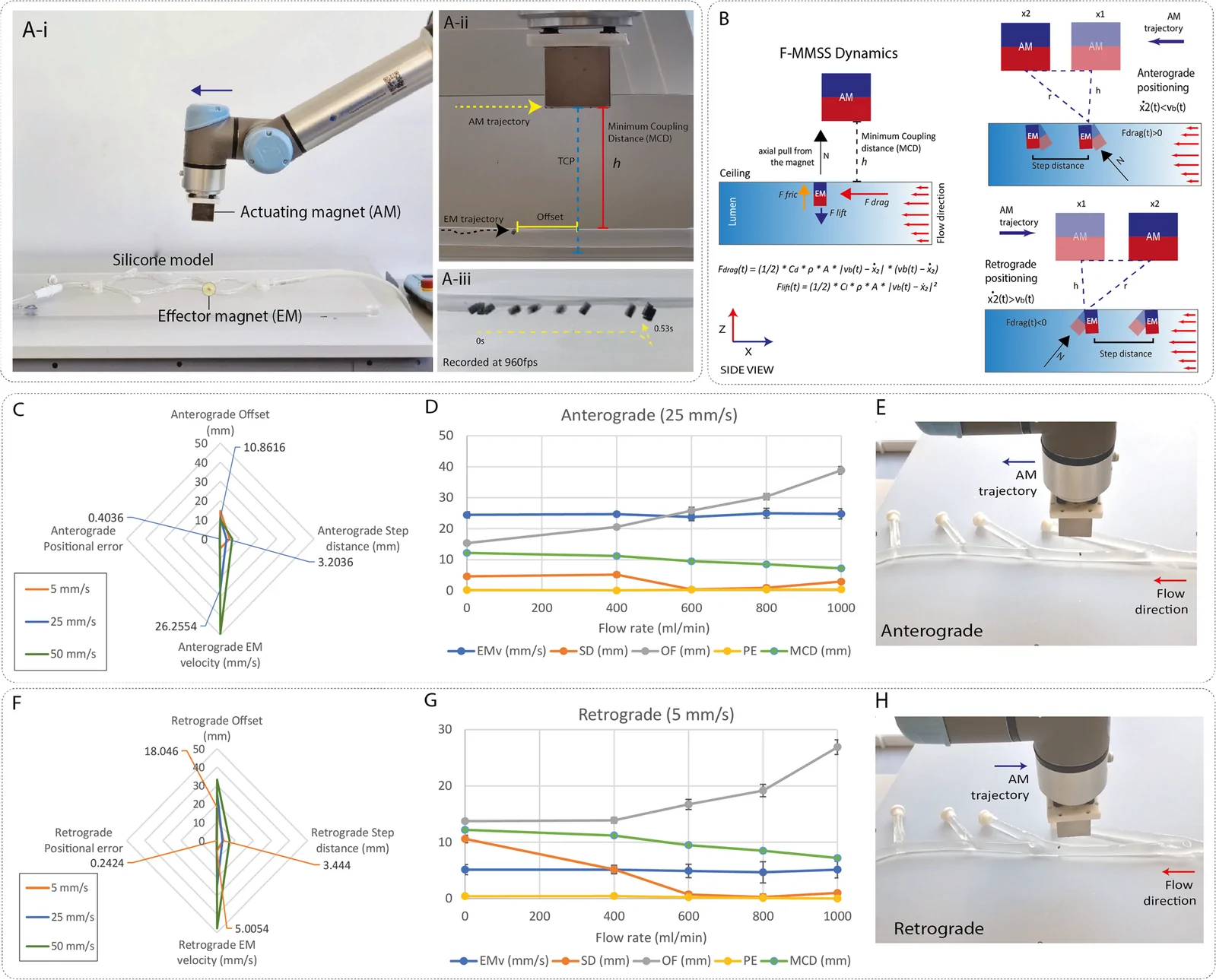
In-vitro magnetic positioning and flow-responsive, magnetically modulated stick-slip. **A)** Experimental setup for magnetic actuation: **(i)** Robotic arm controls an actuator magnet (AM) to drive an internal effector magnet (EM) through a silicone vascular phantom; **(ii)** Minimum coupling distance and relative positioning offset between AM and EM are defined; (**iii)** Time-lapse imaging captures stick-slip motion during navigation. **B)** Schematic representation of F-MMSS dynamics in anterograde and retrograde positioning scenarios. **C**,** F)** Radar plots of EM velocity, offset, step distance and error averaged over all flow rates in anterograde and retrograde conditions respectively. **D**,** G)** Parameter evolution at optimal AM velocities **(**25 mm/s anterograde, 5 mm/s retrograde) across flow rates (0–1000 mL/min), including EM velocity, step distance, offset, positional error and coupling distance. **E**,** H)** Magnetic navigation in anterograde and retrograde guidance respectively demonstrate successful EM positioning using optimized parameters. All experimental measurements were conducted with three independent replicates (*n* = 3) unless otherwise noted. Results are expressed as mean ± st andard deviation

To systematically evaluate positioning accuracy, we developed an in vitro magnetic actuation platform comprising a programmable robotic actuator magnet (AM) and vascular phantom containing the EM (Fig. 3A-i). The AM followed user-defined paths above the phantom, generating time-varying magnetic fields that propelled the EM through the lumen.

We quantified navigation performance using four key metrics: (1) Lateral offset (OF): Distance between AM and EM positions (2) Step distance (SD): Magnitude of discrete EM movements during stick-slip motion (Fig. 3A-iii) (3) Positional error (PE): Cumulative deviation between EM trajectory and intended AM path (Fig. 3A-ii) (4) EM velocity: Speed relative to AM displacement. These metrics were evaluated under both anterograde (flow-assisted) and retrograde (flow-opposed) conditions across flow rates of 0–1000 mL/min.

To ensure that navigation tests accounted for physiologically relevant lumen–device interactions, we characterized frictional properties of the silicone phantom substrates via incline-plane testing and evaluated several lubricants as coatings on the s ilicone to identify conditions that would closely match relevant biological tissue. Silicone coated with a silicone-oil formulation exhibited static and rolling friction values closely matching ex vivo porcine aorta (Supp. Supp Fig. S3-C, D, E. All navigation experiments were therefore performed using silicone phantoms coated with the silicone-oil formulation, which best matched the frictional and morphological characteristics of real vessels. We additionally assessed whether repeated device contact could induce navigation-induced damage in vascular tissue. A reciprocating actuation test on ex vivo porcine aorta showed that repeated capsule motion produced only mild surface compression, evidenced by reduced peak height and stable interfacial area, while confocal 3D topography maps confirmed localized smoothing rather than abrasion, tearing, or other navigation-induced damage (Supp. Fig. S4A, B), indicating that magnetic navigation imposes minimal mechanical impact on vessel walls.

#### Parameter optimization via design of experiments

To identify critical control parameters, we performed a statistically validated Design-of-Experiments (DOE) analysis evaluating twelve system variables (Supp.Table S1, Supp Fig. S3-B). These included magnetic parameters (AM size: 38–80 mm, magnetization directions), mechanical properties (EM weight: 115–300 mg, friction coefficients) and flow conditions (pump rate: 0–150 bpm, viscosity: 0.9–1.6 cP). Statistical analysis using LogWorth scores (− log₁₀p) identified three dominant parameters: (1) Actuation direction (anterograde vs. retrograde); (2) AM geometry (cube size); (3) AM velocity (5–100 mm/s). Coupling distance mapping revealed that larger AMs retained EM control at higher perfusion rates (Supp Fig. S3-A). Confirmatory experiments (*n* = 3) using a 38 mm cube AM revealed optimal velocities were flow-dependent: 25 mm/s for anterograde positioning and 5 mm/s for retrograde positioning (Fig. 3C-G). Successful navigation trials using these optimized parameters are shown in Fig. 3E, H and Supp.Vid. S2.

#### The F-MMSS solution: theoretical framework

**Fig. 4:**
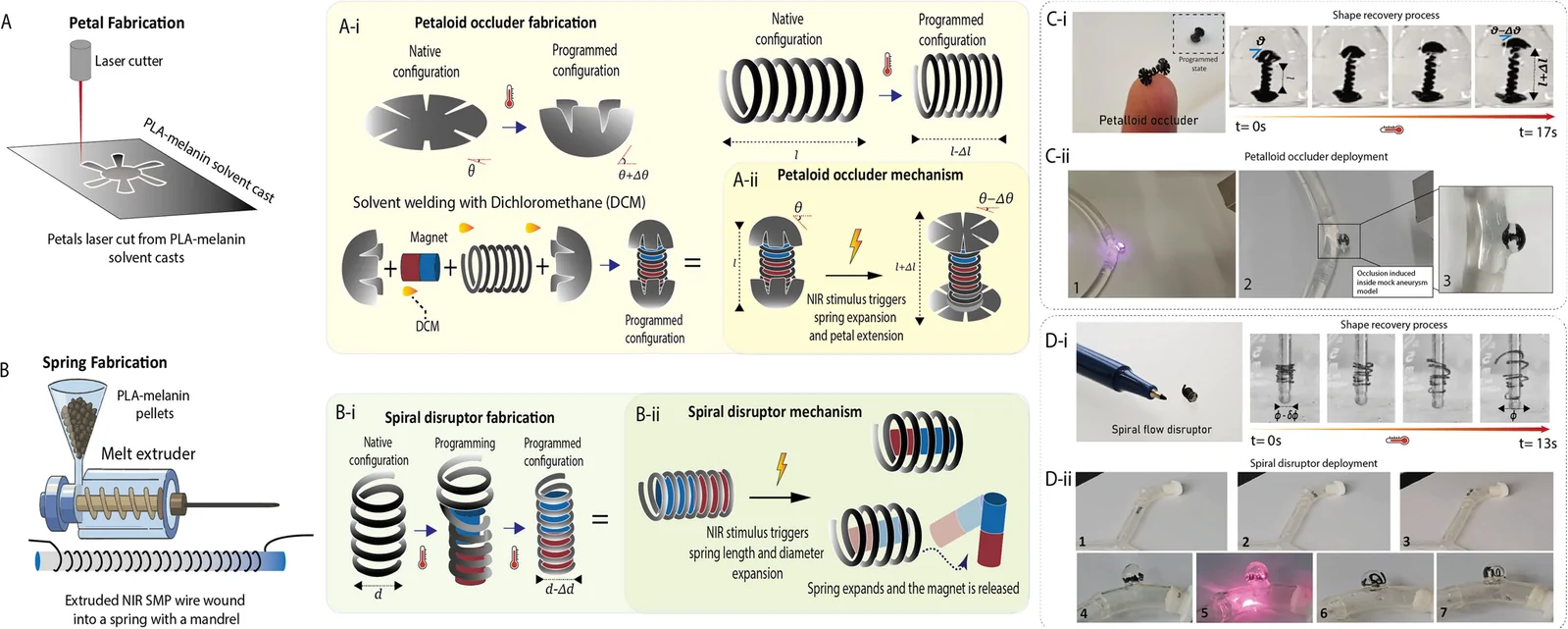
Device fabrication and NIR-triggered deployment. **(A)** Petal elements laser-cut from PLA–melanin casts were assembled into a petaloid occluder by folding and solvent-welding around a central magnet **(A-i).** Upon NIR exposure, petals reopen to restore the programmed shape and exp and within the lumen **(A-ii). (B)** Spiral disruptors were formed by winding extruded PLA–melanin wires, then programmed into compacted geometries and mounted on magnets **(B-i).** NIR activation triggered elongation and magnet release (B-ii). **(C)** In vitro validation of petaloid occluder shows successful manual recovery (i) and NIR-triggered expansion in a vascular phantom (ii). **(D) (i–ii)** Spiral disruptors showed reliable deployment and lumen engagement post-triggering

To explain observed stick-slip transitions (Fig. 4A-iii), we developed the Flow-responsive Magnetically Modulated Stick-Slip (F-MMSS) framework. This model, schematically represented in Fig. 3B, captures the interplay between four time-varying forces acting on the EM:

**Magnetic forces**:

Axial pull: Fₓ(t) = k (x₁ - x₂)/r⁴.

Wall-normal load: F_y_(t) = kh/r⁴.

**Hydrodynamic forces**:

Drag: F_drag_(t) = ½ C_d_ ρ A |vb - ẋ₂|(vb - ẋ₂).

Saffman lift: F_lift_(t) = ½ Cl ρ A |vb - ẋ₂|².

Where k = (µ₀M₁M₂)/(4π) is the magnetic coupling constant, r is the center-to-center distance between the two magnets and vb is blood velocity.

#### Stick-Slip transition criterion and Flow-Dependent control

Stick-slip transitions occur when the driving forces overcome static friction: |Fₓ(t) + F_drag_(t)| ≶ µs N(t).

Where the normal load is N(t) = F_y_(t) − (ρs − ρ) Vg – F_lift_(t).

This framework explains why optimal AM velocities differ between flow conditions: Anterograde flow (vb > ẋ₂): Drag assists motion, requiring higher AM velocity (25 mm/s) to maintain coupling; Retrograde flow (ẋ₂ > vb): Drag opposes motion, necessitating lower AM velocity (5 mm/s) for stable control. The F-MMSS model thus provides critical design guidelines for optimizing shape and control strategies for robust navigation through tortuous vessels. Complete derivations and parametric studies are provided in Supplementary Note S1 and Supp.Fig. S6.

### Device fabrication, in-vitro testing and imaging

Having characterized both the shape memory response and magnetic navigation dynamics separately, we next integrated these capabilities into therapeutic devices. Two distinct architectures were developed to address different clinical scenarios while leveraging the optimized material properties and navigation principles established above. The two NIR-responsive devices were developed using PLA–melanin composites: a petaloid occluder and a spiral disruptor. Device selection was geometry-dependent: petaloid occluders were optimized for narrow-neck aneurysms (≤ 5 mm) to maintain compact navigation profiles while ensuring complete sealing, while spiral disruptors were designed for wide-neck geometries where partial flow modulation is preferred and magnet retrieval remains feasible. Fabrication combined solvent casting, filament extrusion, laser cutting, spring programming and solvent welding (Fig. 4A-B). Based on the 6 wt% melanin optimization from Sect. 2.1, device dimensions were optimized for 3–5 mm vessel diameters: occluders measured 5 × 8 mm (compact) exp anding to 8 × 11 mm (deployed, ± 7.32% SD, *n* = 3), while disruptors measured 4 × 6 mm exp anding to 6 × 15 mm (deployed, ± 11.43% SD, *n* = 3) (Fig. 4A-B). For the occluder, petal-shaped arms were laser-cut from PLA–melanin films and the spring was melt-extruded over a m andrel. These components were shape programmed and bonded around a central NdFeB magnet (rod magnet Ø 2 mm, height 5 mm, Neodymium N45, nickel-plated) using dichloromethane (DCM). Shape programming details are provided in Supplementary Methods. Upon NIR exposure, the shape memory spring re-elongates, causing the petals to flare outward and anchor against the aneurysm neck (Fig. 4A-ii, Fig. 4C-i). For the spiral disruptor, PLA–melanin filaments were coiled around the cylindrical magnet to form a compact spring-loaded construct (Fig. 3B-i). When triggered with NIR light, the spring exp ands axially and radially, dislodging the magnet and leaving behind a flow-disrupting polymer scaffold (Fig. 4B-ii, Fig. 4D). Both devices remained compact during navigation and reliably underwent shape transformation upon NIR stimulation (Fig. 4C-ii, D-ii, Supp.Vid. S1).

**Fig. 5:**
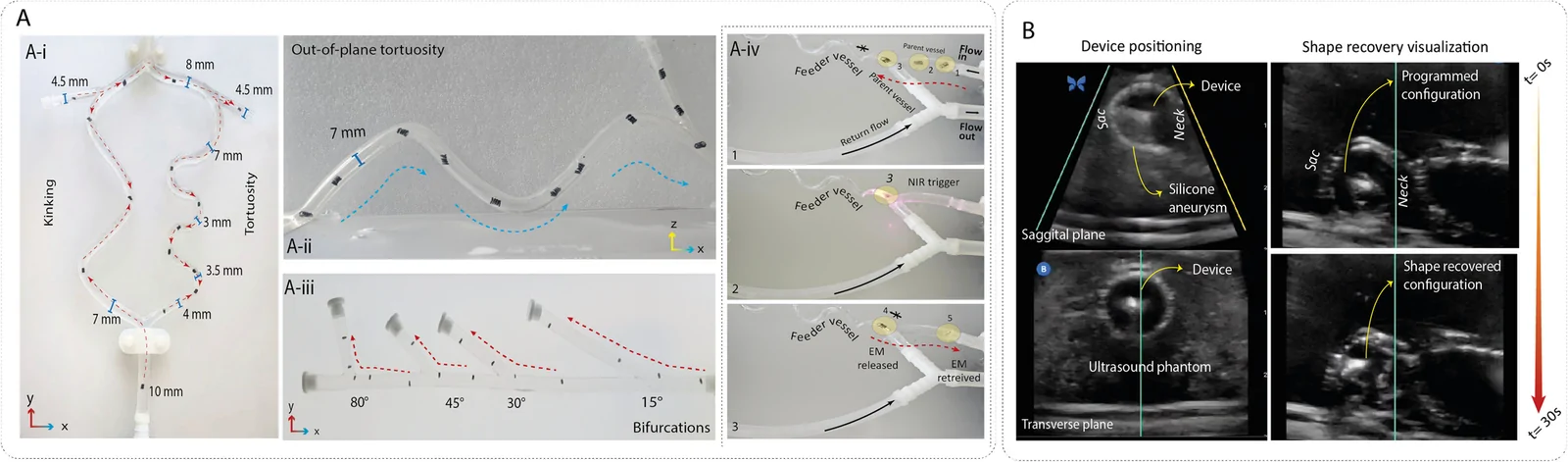
Positioning testing and deployment visualization. **(A)** Magnetic steering was validated in complex phantoms with kinks and bifurcations **(i–iii)** and used to target a feeder vessel for deployment and retrieval **(iv). (B)** Ultrasound-guided positioning and NIR-triggered recovery were demonstrated in an ultrasound phantom

**Fig. 6:**
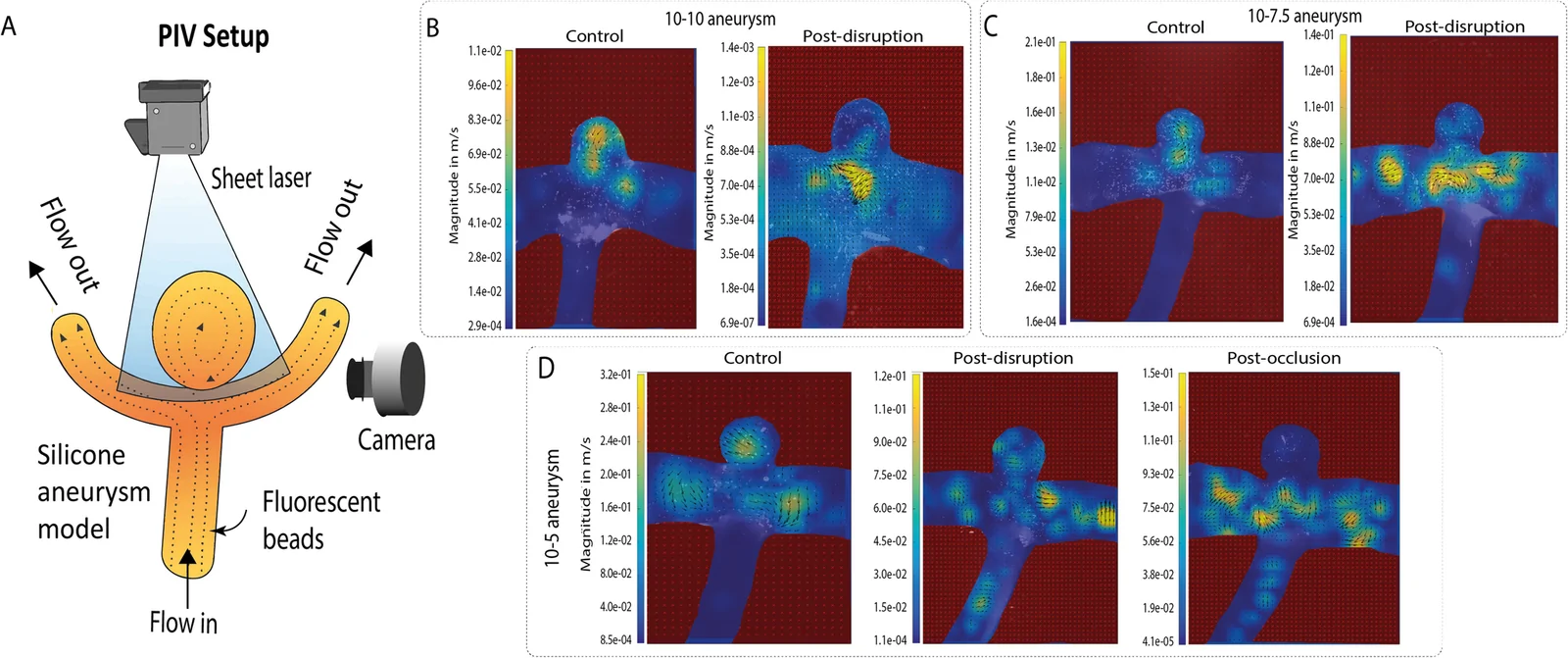
Qualitative hemodynamic analysis of three aneurysm geometries. **A)** PIV setup **B**,** C** and **D)** 10–10, 10 − 7.5, 10–5 mm sac–neck ratio aneurysms respectively and their PIV velocity heatmap comparisons between pristine and disrupted flow conditions

Device positioning was demonstrated via magnetic steering in anatomically relevant silicone phantoms. Trials included tortuous vessels, bifurcations and angulated feeder branches (Fig. 6A i–iii). The petaloid occluder was deployed exclusively in 10 mm sac–5 mm neck aneurysms to ensure secure apposition (Fig. 4C-ii). The spiral disruptor was navigated magnetically into side vessels and deployed via combined steering and NIR triggering (Fig. 4E-iv, Supp.Vid. S3). Ultrasound-guided delivery was validated in aneurysm phantoms, with successful device placement achieved in all cases (*n* = 3). Real-time ultrasound imaging in a phantom provided clear visualization of device navigation and confirmed successful positioning and within aneurysmal geometries prior to NIR activation (Fig. 5B, Supp.Vid. S4). NIR-triggered deployment achieved 100% success rate (*n* = 3) with average deployment times of 35 ± 6 s for occluders and 28 ± 4 s for disruptors through 1 cm tissue phantom.

### Hemoynamics analysis

With functional devices fabricated and successfully positioned, the key question was whether these wirelessly deployed structures are mechanically capable of delivering the intended therapeutic effect. To assess this, we evaluated their hemodynamic impact across multiple aneurysm geometries using a combination of experimental and computational analyses. The hemodynamic effects of the spiral flow disruptor and petaloid occluder were evaluated across three aneurysm geometries (10–10 mm, 10–7.5 mm and 10–5 mm sac–neck ratios) using aneurysm at Y-bifurcation model (Fig. 6A) via particle image velocimetry (PIV) (*n* = 3 per configuration) under pulsatile flow (100 ml/min at 60 bpm) [37]. These aneurysm dimensions were chosen based on clinically relevant aneurysm sac-neck sizes commonly observed in medium-sized arteries [38]. Time-resolved intrasaccular velocity and vorticity analyses obtained by selecting a circular region of interest around the aneurysm sac confirmed that both device types significantly attenuate intra-aneurysmal flow, with geometry-specific performance trends (Fig. 6B-D, Supp.Vid. S5).

**Fig. 7:**
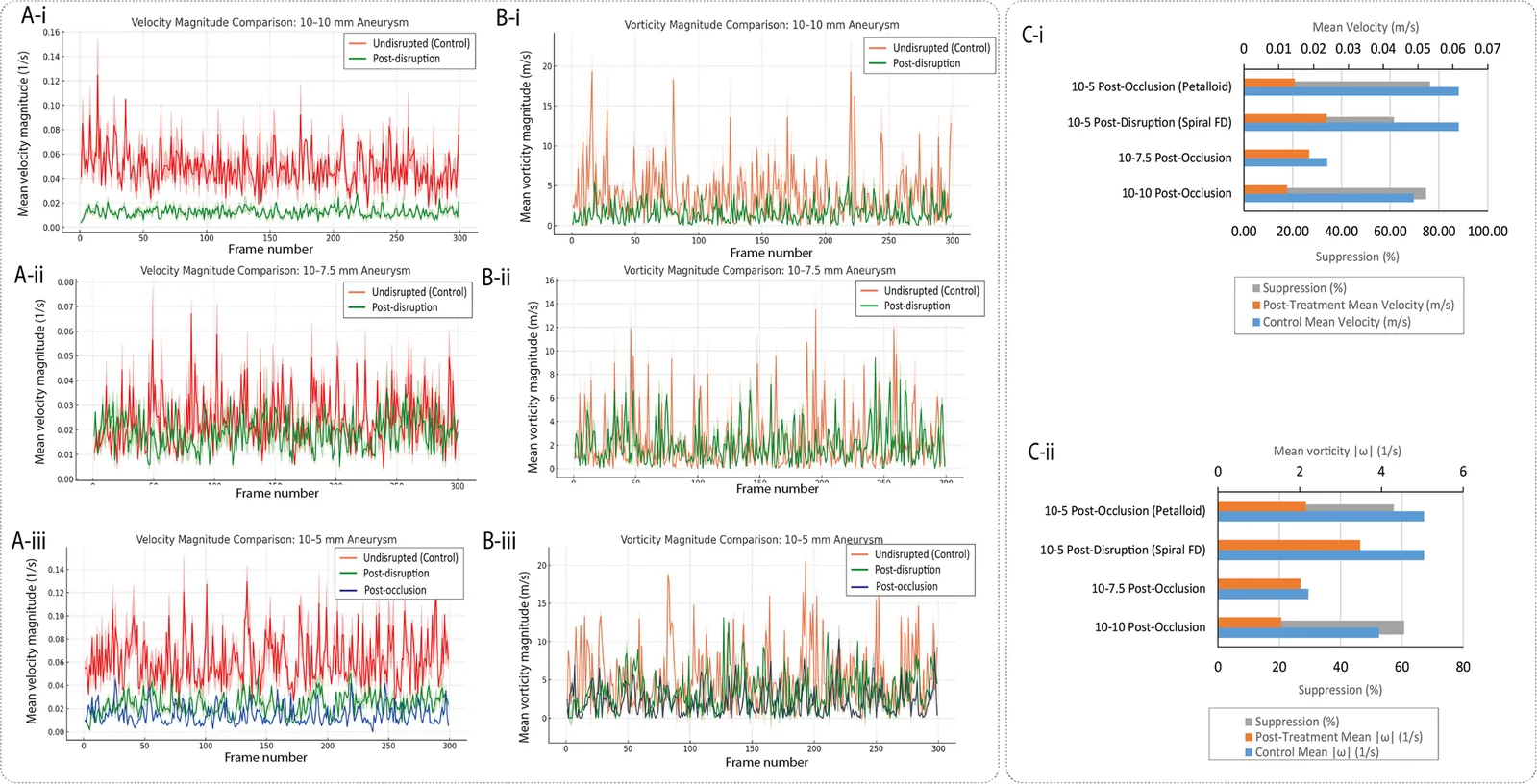
Quantitative Hemodynamic analysis **A i-iii)** Frame-wise intrasaccular velocity magnitude comparison obtained by defining the sac as region of interest of 10–10, 10 − 7.5, 10 − 5 aneurysm respectively **B i-iii)** Frame-wise intrasaccular vorticity comparison of 10–10, 10 − 7.5, 10 − 5 aneurysm respectively **C)** Suppression metrics: **(i)** Mean velocity before and after treatment, with percentage suppression. **(ii)** Mean vorticity and percentage reduction across all conditions. Experiments were replicated three times (*n* = 3) for statistical validity, with exceptions clearly indicated. Data represents mean values ± standard deviation

Spiral disruptors were tested across all geometries, as they are designed for wide-neck aneurysms where complete occlusion is challenging. In 10–10 mm aneurysms, the spiral flow disruptor achieved 74.6% velocity and 60.7% vorticity suppression due to high baseline inflow (Fig. 7A-i, B-i). The 10–7.5 mm geometry showed reduced baseline flow, resulting in moderate suppression (21.5% velocity, 8.7% vorticity) as seen from Fig. 7A-ii, B-ii. In 10–5 mm aneurysms, the spiral disruptor yielded 61.5% velocity and 31.0% vorticity suppression despite only moderate baseline flow levels (Fig. 7A-iii, B-iii).

The petaloid occluder was evaluated in 10–5 mm narrow-neck aneurysms, where its compact pre-deployment footprint enables navigation through small vessels while providing complete neck sealing, after NIR stimulation. In this optimal geometry, the occluder achieved 76.4% velocity and 57.3% vorticity suppression. This geometry-specific deployment strategy highlights that device selection depends on both anatomical constraints and therapeutic goals, spiral disruptors for partial flow modulation in wide necks and petaloid occluders for complete sealing in narrow necks.

To complement PIV, we performed Computational fluid dynamics (CFD) on a representative aneurysm geometry to capture mechanistic features such as wall shear stress (WSS) and inflow impingement visualization which PIV cannot elucidate but are critical predictors of aneurysm rupture risk. CFD of 10 − 5 aneurysm geometry pre and post occlusion or disruption revealed vital streamline visualizations that showed disruption of in-flow jets and vortices (Supp Fig. S5-A), while wall shear stress maps revealed significant reduction at the aneurysm neck post-deployment (Supp Fig. S5-B) supporting the potential for mitigating rupture risk. Quantitatively, sac-averaged WSS decreased from 1.03 N/m² in the unoccluded control to 0.47 N/m² after disruptor deployment and 0.11 N/m² after occluder deployment, demonstrating a 2 to 9-fold reduction in CFD (Supp Fig. S5-C). These reductions in WSS, together with flow stagnation, increased residence time can theoretically create optimal conditions for thrombosis initiation and organization thereby facilitating natural aneurysm flow exclusion and dome stabilization to reduce rupture risk [39–42]. Further validation in biological models with intact coagulation cascades will be necessary in future to establish clinical potential. Together, these results confirm that the NIR-triggered devices effectively modulate flow in both aneurysmal sacs and narrow feeder vessels, with performance governed by the interplay between baseline hemodynamics, geometric fit and device-specific design constraints (Fig. 7C-i, ii). In tumor feeder vessels, the spiral disruptor interrupts inflow and redirects vorticity to the parent artery, increasing flow and rotational energy. This redistributes flow and suggests a mechanism for controlling feeder vessel flow efficiently and effectively (Supp Fig. S7).

### Degradation, biocompatibility and retention

**Fig. 8:**
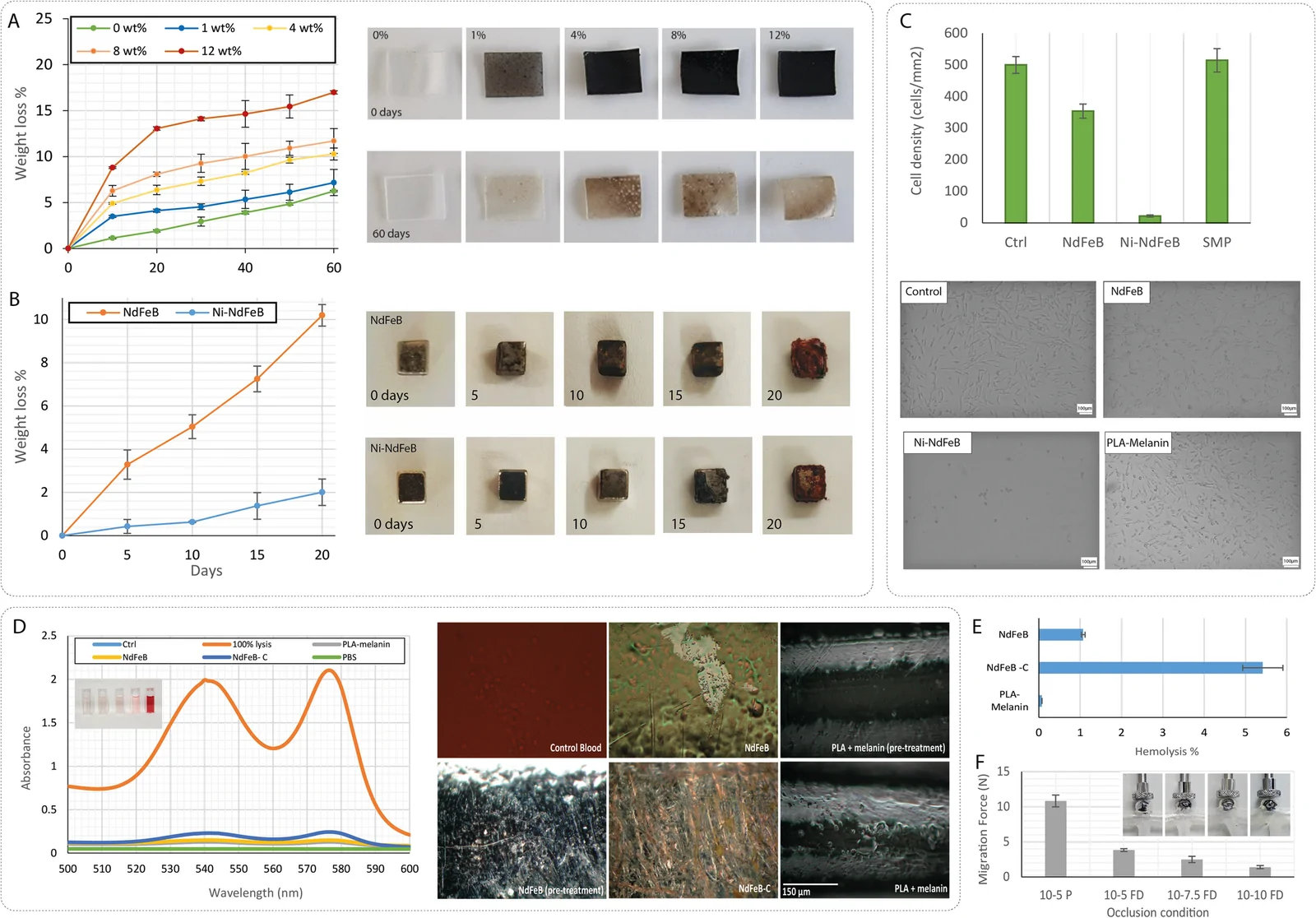
Biocompatibility, corrosion resistance and migration force of PLA–melanin and NdFeB-based devices. **(A)** PLA–melanin films (0–12 wt%) showed gradual degradation in H₂O₂ over 60 days. **(B)** Bare NdFeB corroded severely, unlike Ni-coated NdFeB. **(C)** MEM elution assay revealed reduced cell density on NdFeB surfaces, while PLA–melanin remained cytocompatible. **(D–E)** Hemolysis assays of NdFeB and PLA-melanin **F)** Migration force testing of various disruptor-occluder configurations. All experimental measurements were conducted with three independent replicates (*n* = 3) unless otherwise noted. Results are expressed as mean ± standard deviation

The promising hemodynamic results demonstrated therapeutic potential, but clinical translation requires validation of safety, stability and long-term biocompatibility. We therefore obtained preliminary degradation, hemocompatibility and mechanical retention of the composite devices and their magnetic cores (Fig. 8). Accelerated degradation in H₂O₂ showed melanin-dependent erosion: PLA–melanin films with 12 wt% melanin exhibited > 15% mass loss over 60 days, with darkened, porous residues consistent with bulk degradation (Fig. 8A). Bare NdFeB magnets degraded rapidly, losing > 10% mass in 20 days, while Ni-coated variants showed slower but incomplete protection (Fig. 8B).

Hemocompatibility assays revealed negligible hemolysis for PLA–melanin (0.08%) and a low response for pristine NdFeB (1.07%). Corroded NdFeB exceeded the 5% threshold (5.42%), classifying it as borderline hemolytic (Fig. 8D–E). Microscopy confirmed extensive fouling and corrosion in NdFeB samples, whereas PLA–melanin maintained a clean, non-fouled surface. Cytotoxicity testing using NIH 3T3 fibroblasts further confirmed biocompatibility of PLA–melanin, while corroded NdFeB caused substantial declines in cell viability (Fig. 8C). For long-term implants where magnet retrieval is impractical, NdFeB may be replaced with densely loaded magnetic nanoparticle composites with tunable size-dependent hemocompatibility profiles [43, 44].

Mechanical stability was assessed via migration force testing across different aneurysm neck geometries (Fig. 8F). The 10–5 mm petaloid occluder exhibited the highest anchorage force (10.83 N), attributed to its conformal petal expansion and confined deployment space. Spiral disruptors showed geometry-dependent retention, 3.83 N (10–5 mm), 2.48 N (10–7.5 mm) and 1.39 N (10–10 mm) reflecting reduced constraint in wider-neck aneurysms. These results indicate that retention strength can be tuned through diameter-specific sizing to minimize downstream migration risks under physiological flow.

Collectively, the degradation, hemocompatibility and retention data support the translational feasibility of PLA–melanin devices while identifying NdFeB corrosion as a key engineering challenge for chronic applications.

## Conclusions

This study successfully integrated two previously separate technologies - magnetic microdevice navigation and NIR-triggered shape memory deployment - into a unified platform for wireless endovascular therapy. By coupling a ceiling-constrained AM–EM system with melanin-doped PLA SMPs, we enabled wireless positioning and remote morphing within anatomically realistic phantoms. Two device configurations were developed: a petaloid occluder for complete aneurysm sealing and a spiral disruptor for partial flow modulation in tumor-feeding vessels. Both remained compact during navigation and reliably transformed upon NIR activation. Hemodynamic assessments under pulsatile flow (100 mL/min at 60 bpm) confirmed up to 76% intra-aneurysmal velocity suppression, supported by PIV and CFD analyses. The clinical significance of these flow changes and their correlation with therapeutic outcomes points to the need for future in-vivo investigations. Biocompatibility testing revealed negligible hemolysis (< 1%) for PLA–melanin composites, with retention forces of 1.39–3.83 N expected to prevent downstream embolization.

## Limitations and outlook

Several topics merit attention in future work. This study focuses on distal microvascular navigation and does not yet assess long-range traversal through full-scale vascular structures such as the aortic arch. In envisioned clinical use, the microdevice would be positioned via catheter-assisted proximal placement, in which a tethered delivery tool places the system near the inaccessible section, after which magnetic actuation guides it to the target. Future work will extend the actuation platform to more anatomically extensive vascular pathways and increase sample sizes beyond the current *n* = 3. Current CFD simulations assumed laminar flow but in future, transitional flow modeling may be required for larger vessels or higher flow rates. Further, reliance on flow surrogate metrics without direct thrombosis assessment, evaluation in only a few aneurysm geometries, absence of closed-loop feedback control and lack of ex vivo or in vivo validation are some limitations that warrant future attention. Safety considerations include potential device fragmentation, inflammatory responses to degradation products, long-term magnet fate and MRI compatibility. Synthetic blood analogues lacked a functional clotting cascade, requiring studies in complete hemostatic systems. Priorities include large-animal in-vivo validation, chronic biocompatibility and image-guided closed-loop control. Importantly, this work demonstrates through tetherless microrobotics that inflow reduction alone can increase residence time and promote thrombosis, aligning with established findings in aneurysm hemodynamics [29, 30, 45].Several established aneurysm treatments likewise rely on inflow attenuation, including coiling [46–48], flow-diverting stents [49, 50], intrasaccular devices [51, 52] and surgical clipping [53]. From a microrobotics standpoint, this principle of inducing therapeutic flow states through disruption rather than complete occlusion can enable microdevices to achieve clinically meaningful outcomes despite their small footprint. This effectively multiplies their therapeutic impact beyond what their size would traditionally allow. Together, these advances position tetherless, magnetically guided, NIR-responsive microdevices as a transformative new class of endovascular therapeutics, capable of reaching and treating vascular territories that conventional tools cannot.

## Supplementary Information

Below is the link to the electronic supplementary material.

Supplementary File 1 (DOCX 7.07 MB)

## Data Availability

No datasets were generated or analysed during the current study.

## Materials availability

All data in the main text or the supplementary are available from the authors upon request.
