# Supplementary material for: Magnetically-guided, stimuli-responsive microdevices for endovascular therapy
Source: Biomed Microdevices. 2026 Jan 20;28(1):6. doi: 10.1007/s10544-026-00791-4 (PMC13652879; doi:10.1007/s10544-026-00791-4)
Supplement: Supplementary file 1 [file 10544_2026_791_MOESM1_ESM.docx]

**SUPPLEMENTARY INFORMATION**

**Contains:**

**Supplementary table S1-S3**

**Supplementary notes S1**

**Supplementary Figures S1-S7**

**Extended Materials and Methods**

**Supplementary videos S1-S5**

**Video captions**

Supp.Vid. S1: Shape recovery behaviour of NIR-SMP structures and devices

Supp.Vid. S2: Effector magnet anterograde and retrograde positioning under flow conditions

Supp.Vid. S3: Magnetic positioning and NIR triggered deployment of microdevice

Supp.Vid. S4: Ultrasound-assisted microdevice deployment

Supp.Vid. S5: Flow visualization with tracers for aneurysms of various sac-neck ratios

**Supplementary Table S1.** Experimental conditions used for characterizing magnetic positioning under varying actuation parameters. A constrained fractional factorial design was employed, sampling combinations of actuator magnet (AM) geometry, effector magnet (EM) properties, lumen characteristics and flow. Some combinations, such as high cardiac output (4000 cc/min) with large AM–EM separation or altered AM magnetization direction, were physically unviable due to EM washout or decoupling. Parameters were hence adjusted as needed to ensure.to ensure viability.

**Supplementary Table S2**. Comparison of existing endovascular technologies and the proposed system

| **Class / Example** | **What it Demonstrates** | **Key Limitations (relative to our device)** |
| --- | --- | --- |
| **SMA / self-expanding vascular stents** | Self-expanding upon temperature or body-temperature, used clinically as stents / vascular implants. Good biocompatibility, stable metallic scaffold.^8,9^ | • Deployment still requires catheter-based delivery  • Once expanded, no remote actuation or reconfiguration • Limited to structural support (not dynamic morphing or retrieval)  • Hard to adapt to non-standard geometries or need for re-shaping |
| **Magnetically actuated guidewire/microcatheter-based probes** | Use external magnetic fields to steer guidewires / microprobes into complex vasculature for catheter-based interventions, reducing mechanical push/pull forces at the tip.^10,11^ | • Still catheter / tether-based, not a fully untethered, deployable system  • Provide steering, but not morphological transformation suitable for occlusion or flow disruption • Often limited to drug delivery or navigational guidance rather than structural intervention |
| **Research-stage magnetic microrobots (untethered, magnetically actuated)** | Demonstrated wireless navigation / control inside fluidic / vascular-mimicking environments; potential for in vivo cargo delivery, targeting, navigation in tortuous vessels.^12–14^ | • Mostly designed for locomotion or cargo delivery, no deployable/reconfigurable structural transformation  • Lack of morphological expansion / shape change (so cannot occlude or modulate flow)  • Many rely on bulky electromagnetic coil setups, limiting clinical practicality  • Payload/biocompatibility constraints; rarely tested for flow-modulation or occlusion |

**Supplementary Table S3.** Comparison of Standard Aneurysm Treatments and F-MMSS Microdevices

| Modality | Delivery Method | Mechanism | Suitable For | Key Advantages | Key Limitations |
| --- | --- | --- | --- | --- | --- |
| **Coiling**^46–48^ | Catheter + guidewire | Intrasaccular packing → thrombosis^46^ | Narrow-neck; proximal/mid vessels^48^ | Long clinical track record; flexible deployment^47^ | Poor wide-neck durability; catheter access limits; recanalization |
| **Flow-Diverting Stents**^49,50^ | Catheter-delivered braided stent | Neck-level flow diversion | Wide-neck sidewall; 2–5 mm parent vessels^49^ | High occlusion rates; single implant^50^ | Risk of in-stent thrombosis, stenosis, or delayed aneurysm rupture; requires dual antiplatelet therapy^49^ |
| **Intrasaccular Devices (WEB/Contour)** ^51,52^ | Catheter into sac | Neck flow disruption^52^ | Wide-neck bifurcations^51^ | No antiplatelets; single-step deployment^51^ | Size-dependent performance; incomplete occlusion; catheter access required |
| **Surgical Clipping**^53^ | Open craniotomy | Mechanical neck closure | Accessible proximal aneurysms | Immediate, durable cure; no antiplatelets | Highly invasive; long recovery; limited deep/posterior access |
| **F-MMSS Microdevices (This Work)** | Tetherless magnetic navigation + NIR-triggered SMP | Morphing occluder or flow disruptor | Distal, tortuous, small-calibre vessels | Navigation beyond catheter limits; wireless deployment; polymer-only option | In vitro only, requires magnetic + NIR setup; long-term biocompatibility untested |

**Supplementary Fig. S1: Thermal and mechanical characterization of PLA–melanin composites.
A)** DSC curves show consistent Tg across 0–12 wt% melanin. **B)** TGA confirms thermal stability up to ~230 °C with similar onset degradation. **C)** Stress–strain curves reveal increasing stiffness with melanin loading (20–40 MPa ultimate strength), highlighting the trade-off between reinforcement and processability.

**Supplementary Fig. S2 NIR-Responsive Shape Memory Polymer (SMP) beam characterization. A)** Photothermal conversion efficiency of PLA-melanin as a function of melanin concentration. **B)** Shape recovery of a bent beam under load via NIR, with corresponding force per unit volume plotted for different melanin contents

**Supplementary Fig. S3**. **Magnetic positioning parameters and lubricant testing A)** Design of experiments identifies key parameters affecting magnetic guidance-AM velocity, actuation direction and geometry based on their impact on EM responses - velocity, positional error, offset and step distance. **B)** Minimum achievable coupling distance plotted across AM geometries and flow rates. **C)** Peak density and average peak height of surface asperities measured by laser confocal microscopy on uncoated silicone, lubricant-coated silicone and ex vivo porcine aorta. **D)** Static and rolling coefficients of friction measured using an incline-plane tester across uncoated silicone, ex vivo aorta and silicone coated with Ease Release 200 (wax-based) or Go On Spray (silicone-oil-based). **E)** Representative 3D confocal height maps and corresponding line-profile sections showing the progression of surface roughness from smooth silicone to lubricant-coated silicone and ex vivo aorta.

**Supplementary Fig. S4. Mechanical Impact of magnetic navigation on ex-vivo tissue. A)** The device was actuated over ex-vivo tissue in a linear reciprocating motion, with each forward and backward pass counted as one cycle. These trends collectively indicate that repeated device motion induces surface flattening and peak reduction consistent with microstructural compression, rather than material removal or abrasive damage. The stable developed interfacial area further supports the absence of contact-induced surface texturing or tearing. **B)** Confocal topography maps of the abraded region across 5 cycles show gradual loss of micro texture and topographical flattening. Each heatmap reflects 3D surface deformation localized to the abrasion path.

**Supplementary Fig. S5: CFD analysis of aneurysm geometries. A)** CFD obtained velocity field streamlines showing: **i)** Control case (unmodulated vortex), ii) Post-disruption (spiral disruptor), **iii)** Post-occlusion (petaloid occluder), with observable collapse of intra-saccular flow. **B)** Wall shear stress maps for control, spiral and petaloid devices showing reduction in peak neck shear. **C)** CFD-based Wall shear stress measurement showing WSS reduction post-disruption and post-occlusion

**Supplementary notes**

**S-1 Flow-responsive Magnetically Modulated Stick-Slip (F-MMSS)**

The Flow-responsive Magnetically Modulated Stick-Slip (F-MMSS) regime describes the motion of a magnetically guided capsule (effector magnet, EM) under the influence of a moving external actuator magnet (AM) in the presence of pulsatile blood flow. Unlike dry-lumen systems, the F-MMSS regime accounts for hydrodynamic drag and Saffman lift forces arising from blood flow, along with magnetically modulated axial and normal forces as shown in Supp Fig. S3.

**Supplementary Fig.S6. Flow-responsive Magnetically Modulated Stick-Slip (F-MMSS) dynamics schematic.** Illustration of the F-MMSS framework showing four forces acting on the effector magnet (EM): axial magnetic pull (F_x_), wall-normal load (F_y_), hydrodynamic drag (F_drag_) and Saffman lift (F_lift_). Left panel shows geometric parameters and force vectors in side view. Right panels compare anterograde positioning (top, flow assists motion) versus retrograde positioning (bottom, flow opposes motion), with corresponding mathematical expressions for drag and lift forces based on relative velocity between blood flow and EM motion.

## 1. Geometric Basis and Magnetic Force Derivation

The magnetic interaction between the AM and EM depends on their relative axial and vertical positions:
x_1_(t): axial position of the actuator magnet (AM)
x_2_(t): axial position of the effector magnet (EM)
h: fixed vertical separation between AM and EM
r(t): Euclidean distance between AM and EM, given by:
 r(t) = √ ((x_1_ - x_2_)^2^ + h^2^)
The total magnetic attraction force between AM and EM follows:
 F_total_ = k / r^3^, where k = (μ_0_ M1 M2)/(4π)
This total force is resolved into:
- Axial (driving) force:
 F_x_(t) = k(x_1_ - x_2_)/r^4^
- Wall-normal (normal load) force:
 F_y_(t) = kh/r^4^

## 2. Inclusion of Buoyancy-Corrected Weight

In submerged environments, the net weight experienced by the capsule is reduced by buoyant forces. Hence, we replace the gravity term mg with:
 W - B = (ρ_s_ - ρ) V g
Where:
ρ_s_: density of the EM/device material
ρ: density of the fluid (blood)
V: volume of the capsule
g: gravitational acceleration
This correction ensures accurate modeling of the capsule’s wall contact force in the vessel, especially for materials with densities close to blood.

## 3. Hemodynamic Forces

Under pulsatile blood flow, the EM experiences:
- Drag Force (resists or assists motion relative to flow):
 F_drag_(t) = 1/2 C_d_ ρ A |vb(t) - ẋ_2_(t)| (vb(t) - ẋ_2_(t))
This formulation ensures the drag force always opposes the relative motion where vb(t) = blood velocity as a function of time.
**Saffman Lift Force (shear-induced, reduces contact):**
F_lift_(t) = 1/2 C_l_ ρ A |vb(t) - ẋ_2_(t)|²
This vertical force arises from the parabolic velocity profile of laminar blood flow and acts to lift the EM toward the lumen center, reducing friction during systolic acceleration.

## 4. Normal Contact Force

The total contact load governing friction is:
 N(t) = F_y_(t) - (ρ_s_ - ρ) Vg - F_lift_(t)
This value dictates how strongly the EM presses against the lumen wall.

## 5. Stick-Slip Transition Criterion

The stick-slip transition is governed by the inequality:
 |F_x_(t) + F_drag_(t)| ≶ μ_s_ N(t)
Substituting full expressions: |k(x_1_ - x_2_)/r^4^ + 1/2 C_d_ ρ A |vb(t) - ẋ_2_(t)| (vb(t) - ẋ_2_(t))| > μ_s_ [kh/r^4^ - (ρ_s_ - ρ)Vg - 1/2 CL ρ A |vb(t) - ẋ_2_(t)|²]
In anterograde flow (vb(t) > ẋ_2_(t)), drag assists motion, lowering the slip threshold. In retrograde flow (ẋ_2_(t) > vb(t)), drag resists motion, increasing the required magnetic pull.
These relationships provide a theoretical framework for tuning of AM trajectory, EM design and surface friction for vascular navigation.

**CFD and PIV for tumor feeder vessels**

CFD and PIV validation in a feeder vessel model (Fig. 7A-i, ii respectively) demonstrated that the spiral disruptor interrupted the inflow and redirected vorticity into the parent artery, with quantifiable increase in flow and rotational energy in parent vessel indicating a redistribution of flow, thereby suggesting a potential mechanism for feeder vessel flow control (Fig. 7A-iii, iv).

**Supp Fig.S7. Hemodynamic analysis of feeder vessel geometry A)** CFD and PIV analysis of feeder vessel disruption: **i)** CFD streamlines before and after spiral disruptor deployment. **ii)** Corresponding velocity heat maps. **iii–iv)** Time-resolved velocity and vorticity comparison in parent and feeder vessels. **B)** Suppression metrics: **i)** Mean velocity before and after treatment, with percentage suppression. **ii)** Mean vorticity and percentage reduction across all conditions.

**Extended Materials and Methods**

**3.1. Materials and Device Fabrication**

**3.1.1. PLA-Melanin Composite Preparation**

Poly(lactic acid) (PLA, MW ~150,000, Sigma-Aldrich) was combined with synthetic melanin (CAS Number: 8049-97-6, Sigma-Aldrich) at concentrations ranging from 0-12 wt%. Films were prepared via solvent casting using dichloromethane (DCM, 99.8%, Sigma-Aldrich) as the solvent. PLA pellets and melanin powder were dissolved in DCM at 10% w/v concentration, stirred for 24 hours at room temperature and cast into Petri dishes. Films were dried under ambient conditions for 48 hours followed by vacuum drying at 40°C for 12 hours to ensure complete solvent removal. For filament fabrication, PLA-melanin composites were melt-extruded using a 3devo Maker ONE filament maker at 180-200°C with a 1.75 mm die. Extrusion parameters were optimized for each melanin concentration to ensure consistent diameter and surface finish.

**3.1.2. Magnetic Components**

Neodymium iron boron (NdFeB) permanent magnets were used as effector magnets (EM). Rod magnets (Ø 2 mm × 5 mm, Grade N45, nickel-plated, Supermagnete GmbH and KJ Magnetics Inc.) served as the primary magnetic cores. Actuator magnets (AM) consisted of NdFeB cube magnets ranging from 38-80 mm edge length (Grade N52, nickel-plated, Supermagnete GmbH and KJ Magnetics Inc.). Magnetization directions were specified as either axial or diametric based on experimental requirements.

**3.1.3. Device Assembly**

Petaloid Occluder: Petal-shaped arms were laser-cut from PLA-melanin films using a CO₂ laser (Universal Laser Systems VLS2.30) at optimized power and speed settings. Spring components were melt-extruded over stainless-steel mandrels and shape-programmed by compression at 70°C followed by cooling under constraint. Components were assembled around the central NdFeB magnet using dichloromethane (DCM) as a solvent weld, ensuring secure bonding while maintaining shape memory functionality.

Spiral Disruptor: PLA-melanin filaments were wound around cylindrical NdFeB magnets using a custom winding apparatus. The assembly was then compressed and heated to 65°C for shape programming, creating a compact configuration that expands upon NIR activation.

**3.2. Shape Memory Characterization**

**3.2.1. Thermal Analysis**

Differential scanning calorimetry (DSC) and thermogravimetric analysis (TGA) were performed using a Linseis TGA/DSC PT1600 under nitrogen atmosphere with heating rates of 10°C/min from 25-150°C to determine glass transition temperatures and evaluate thermal stability from 25-600°C.

**3.2.2. Mechanical Testing**

Tensile testing was conducted using an Instron 5942 universal testing machine with a 100 N load cell at 5 mm/min crosshead speed on ASTM D638 Type IV specimens. Young's modulus was calculated from the linear region of stress-strain curves.

**3.2.3. Shape Memory Evaluation**

Shape fixity (Rf) and shape recovery (Rr) were quantified using standard protocols. Fixity was calculated as Rf = (θf / θm) × 100%, where θf is the fixed angle and θm is the maximum deformation angle. Recovery was determined as Rr = (θr / θm) × 100%, where θr is the recovered angle. Recovery kinetics were measured under NIR irradiation (808 nm laser, adjustable power 0.5-2 W/cm²) with thermal imaging (FLIR TG165-X) for temperature monitoring.

**3.3. Magnetic Actuation System**

**3.3.1. Experimental Setup**

A 6-axis robotic arm (Universal Robots UR5e) positioned the actuator magnet above silicone vascular phantoms. The phantom was mounted in a closed-loop flow circuit with a cardiovascular waveform pump (Model 1407, Harvard Apparatus) providing pulsatile flow at rates of 0-1000 mL/min. Flow waveforms mimicked physiological cardiac cycles at 60-150 bpm.

**3.3.2. Vascular Phantoms**

Silicone vascular phantoms were purchased from Ningbo Trando 3D Medical Technology Co., Ltd. (Zhejiang, China). The phantoms featured Shore hardness values between 30A and 60A to simulate different vessel compliance. Internal geometries included straight tubes (3-5 mm diameter), bifurcations, kinks, tortuosities and aneurysm models with various sac-to-neck ratios of 10:10, 10:7.5 and 10:5 mm.

**3.3.3. Navigation Metrics**

Three positioning metrics were tracked via video recording and analyzed using Physlets Tracker software through a combination of manual keyframing and autotracking. Lateral offset (OF) was measured as the distance between the tool center point of AM and the point where the EM touches the lumen ceiling. Step distance (SD) quantified the magnitude of discrete EM movements during stick-slip motion. Positional error (PE) was calculated as the z-score between the distributions of the y-coordinates of AM trajectory and EM trajectory using the formula z=(x̄₁-x̄₂)/√((σ₁²)/n₁ + (σ₂²)/n₂), where x̄₁ is the mean of the first sample (AM), x̄₂ is the mean of the second sample (EM), σ₁² is the variance of the first sample (AM), σ₂² is the variance of the second sample (EM) and n₁ and n₂ are the sample sizes of AM and EM respectively.

**3.4. Lumen friction and surface topography characterization**

Static and rolling coefficients of friction between the device and lumen surfaces were measured with a custom adjustable incline-plane tester. A cylindrical surrogate of the device was placed on an inclined strip of either silicone phantom, lubricant-coated silicone, or ex vivo porcine aorta.Two commercial lubricants were evaluated: Ease Release 200 (a wax-based release agent) and Go On Spray (a silicone-oil–based lubricant). The plane angle θ was gradually increased until motion initiated, giving the static friction coefficient μₛ = tanθ_crit. For rolling friction, the object was released or given a small initial velocity and its motion recorded to extract acceleration *a*. The rolling coefficient was calculated as μᵣ = (sinθ – a/g)/cosθ; when *a* ≈ 0, μᵣ ≈ tanθ. Each surface condition was tested in triplicate (n = 3).

The same samples were characterized using laser confocal microscopy () to quantify peak density and average peak height (Fig. S3 C-E). Lubrication with Go On Spray au Silicone shifted the silicone phantom’s friction coefficient and surface asperity distribution into the ex vivo porcine aorta range; therefore, this formulation was used in all subsequent magnetic navigation trials.

**3.5. Design of Experiments (DOE)**

A fractional factorial design evaluated 12 system parameters: AM size (38-80 mm), EM weight (115-300 mg), coupling distance (50-100 mm), AM velocity (5-100 mm/s), magnetization directions (axial/diametric), AM height (0.6-1.6 mm), pump rate (0-150 bpm), fluid viscosity (0.9-1.6 cP), lumen hardness (Shore 30A/60A), friction coefficient (0.6-1.6) and flow direction (anterograde/retrograde). Statistical analysis used JMP Pro 16 with LogWorth scores (−log₁₀p) to identify significant factors. Follow-up one-factor-at-a-time (OFAT) experiments (n=3) validated key findings.

**3.6. Hemodynamic Analysis**

**3.6.1. Particle Image Velocimetry (PIV)**

Flow visualization used PIV with fluorescent green polyethylene microspheres (155-175 μm, 1.025 g/cc, Cospheric LLC) suspended in blood analogue (60% glycerol-water, viscosity 3.5 cP). Illumination sheet was provided by a HUEPAR Purple Line laser Tool. Image acquisition used a camera at 240 fps. Velocity fields were calculated using adaptive correlation algorithms with 32×32 pixel interrogation windows and 75% overlap in PIVLab (v3.08, standalone version) open-source PIV software from Optolution^34^.

**3.6.2. Computational Simulations**

Computational fluid dynamics (CFD) simulations were conducted using COMSOL laminar flow module. Geometries were meshed with tetrahedral elements (element size 0.1 mm near walls, growth rate 1.2). Blood was modelled as a Newtonian fluid (density 1060 kg/m³, viscosity 3.5 mPa·s). Simulations used convergence criteria of 10⁻⁶ for continuity and momentum equations. A three-level mesh refinement study (coarse, normal and fine) was conducted to assess mesh independence. Sac-averaged velocity and sac-averaged wall shear stress (WSS) were used as convergence metrics. Across the three meshes, sac-averaged velocity varied by ±5.83–6.41% and sac-averaged WSS by ±4.82–6.60%. As the qualitative flow structures and device-induced suppression trends were unchanged between the normal and fine meshes, the normal mesh was used for all reported simulations.

**3.6.3. Flow Analysis**

Intrasaccular velocity and vorticity were quantified by defining circular regions of interest, encompassing the aneurysm sac. Temporal averaging over 3 cardiac cycles provided mean values for pre- and post-deployment comparisons. Suppression percentages were calculated as: ((Baseline - Post-deployment)/Baseline) × 100%.

**3.7. NIR Triggering and Deployment**

NIR activation used an 808 nm laser diode (RLTMDL-808-10W, 10 W cw, Roithner Lasertechnik GmbH) with adjustable power density (0.5-10 W/cm²). Tissue penetration studies employed ex vivo chicken tissue at 1 cm thickness. Temperature monitoring during activation used infrared thermography (FLIR TG165-X). Deployment success was defined as >80% shape recovery within 60 seconds of NIR exposure.

**3.8. Biocompatibility and Safety Assessment**

**3.8.1. Hemocompatibility Testing**

Hemolysis assays followed ASTM F756-17 standards. Citrated whole blood (10 mL) was incubated with sample materials (1 cm² surface area) for 3 hours at 37°C with gentle rotation. Hemoglobin release was quantified spectrophotometrically at 540 nm, with hemolysis percentage calculated relative to positive (distilled water) and negative (saline) controls.

**3.8.2. Cell Viability Testing**

Cytotoxicity was evaluated using NIH 3T3 fibroblasts via MEM elution assays (ISO 10993-5). Cells were seeded at 5×10⁴ cells/mL and exposed to material eluates for 48 hours. Viability was assessed through visual grading based on cell confluence compared to control conditions

**3.8.3. Degradation Studies**

Accelerated degradation used 30% hydrogen peroxide to simulate oxidative physiological conditions. Samples were weighed at regular intervals over 60 days, with mass loss percentage calculated. Surface morphology changes were documented using optical microscopy.

**3.8.4. Migration Force Testing**

Retention forces were measured using a custom tensile testing apparatus. Deployed devices were subjected to increasing axial loads until dislodgement occurred. Tests were performed in triplicate for each aneurysm geometry, with maximum force recorded as the migration resistance.

**3.9. Statistical Analysis**

All experiments were performed in triplicate (n=3), unless specified otherwise. Data are presented as mean ± standard deviation.
